# Slow public health: a temporal-relational framework for migration and aging in the Global South

**DOI:** 10.3389/fpubh.2026.1900909

**Published:** 2026-08-14

**Authors:** Miroslava Tokovska, Jozef Suvada

**Affiliations:** 1 Department for Health and Exercise, Faculty of Health Sciences and Technology, Kristiania University of Applied Sciences, Oslo, Norway; 2 Research Department, St. Elizabeth University of Health and Social Work, Bratislava, Slovakia

**Keywords:** aging, asset-based community development, Global South, life-course perspective, migration, slow public health, temporal fit

## Abstract

Population aging and migration represent two of the most profound demographic transformations shaping the Global South. Conventional public health approaches have typically relied on standardized interventions and measurable outcomes, yet such frameworks often fail to capture the temporal, relational, and contextual dimensions affecting aging populations. This perspective introduces “Slow Public Health” as a temporal-relational framework for public health practice in migration and aging contexts. Building on the Slow Movement, Slow Medicine, life-course thinking, and asset-based community development, it emphasizes longitudinal engagement, community capability, and appropriately paced action—not delayed action—rather than rapid but short-lived interventions. The framework recognizes that trust, adaptation, and community participation take time, while urgent risks demand swift responses. It also highlights temporal depth, relational continuity, and community wisdom in regions affected by youth out-migration and rapid demographic aging.

## 1 Introduction

Questions of sustainability have become increasingly central to contemporary public health. Progress in science and public health continually raises new questions. Sustainability, however, does not always require novelty; it may also require a return to overlooked principles and practices. Despite unprecedented technological sophistication and medical capabilities, populations worldwide continue to face mounting challenges related to stress, inequality, and environmental degradation that conventional approaches seem ill-equipped to address (1).

The Global South stands at a particularly critical juncture. The combination of youth migration and rapid population aging creates a demographic landscape characterized by discrepancy and duality. As younger generations migrate to developed nations seeking economic opportunities, older adults are left behind to navigate care provision, emotional support, and wellbeing with diminishing traditional support structures. The growing participation of women in the workforce further disrupts caregiving roles that have historically anchored family and community care systems. These transformations demand not merely new interventions, but a fundamental reconsideration of how public health temporality, relationships, and outcomes are conceptualized (2).

This paper argues that sustainable responses to migration and aging may lie not in accelerating responses, but in deliberately slowing them. “Slow Public Health” is introduced here as a temporal-relational framework that reorients public health practice toward deliberate, thoughtful system-oriented engagement with communities and individuals across the life course. This is not a call for inaction, but rather an invitation to reconsider the elements of effective, sustainable, and humane public health practice.

## 2 The slow movement and its extension to medicine

The Slow Movement emerged in response to the acceleration of modern life. Its roots lie in Slow Food movement in Italy during the 1980s, a protest against fast food culture and its associated values of speed, standardization, and efficiency (3). This movement later expanded into Slow Cities, Slow Fashion, and Slow Education, each prioritizing quality over quantity, presence over productivity, and sustainability over short-term gains.

Slow Medicine, founded in Italy in 2011, applies these principles to healthcare (3). Built upon three pillars (measured action, respectful engagement, and equitable access) Slow Medicine challenges the assumption that more intervention necessarily yields better outcomes. The “Doing more does not mean doing better” campaign in Italy exemplifies this approach, reducing unnecessary tests and treatments through physician-patient dialogue (3, 4). Crucially, the term “slow” signifies deliberate, thoughtful, and system-oriented practice rather than inactive or unresponsive care (5). These principles align with critiques of what scholars term the “toxic culture” of modern health, a culture that has made health and physical fitness into a modern fixation while simultaneously producing conditions that undermine wellbeing through increasing stress, inequality, and environmental degradation (1).

## 3 Toward a slow public health framework

Extending these principles to the population level, Slow Public Health embraces several fundamental orientations that distinguish it from conventional approaches. First, it adopts a life-course perspective that recognizes health as developing across lifetimes rather than through discrete interventions (6). This perspective acknowledges that older adults carry decades of lived experience, and that current health practices must be understood through life history and biographical time. The Developmental Origins of Health and Disease concept demonstrates how factors throughout the life-course determine health outcomes across the lifespan (7).

Multiple temporal dimensions require attention within this framework, beginning with “chronos” itself, time understood not merely as measurable quantity but as quality and depth. Biographical time recognizes that identity change and habit formation proceed slowly at older ages, respecting the gradual nature of transformation rather than expecting rapid behavior change. Cohort time acknowledges that different generational experiences, including war, migration, and economic shifts, shape aging experiences and preclude one-size-fits-all approaches. Community time attends to complex interlocking temporalities including colonial displacement, ecological degradation (2), and unequal access to health technologies in the Global South.

Second, Slow Public Health emphasizes longitudinal engagement, viewing communities not as “intervention sites,” but as partners in health creation. This requires what practitioners of Slow Nursing have termed “being in the moment” (8): deep listening sessions before program design, ethnographic approaches to understand daily rhythms and needs, and dedicated time for trust-building, particularly with marginalized older adult populations (9, 10).

Third, Slow Public Health exercises patience with outcomes, allowing time for community capacity-building and systems change. This contrasts with the project-cycle temporality that dominates conventional public health, where short funding cycles often prevent sustainable community development. The principle of “doing one thing at a time” avoids implementing multiple interventions simultaneously, instead allowing initiatives to take root before expansion (11). Funders could introduce staged financing mechanisms that support process indicators in early phases and health outcomes in later phases, rather than demanding both within short reporting cycles.

Fourth, Slow Public Health recognizes “creating joy and contentment” as legitimate health outcomes. At the population level, this translates to programs that enhance rather than extend quality of life, and recognition that community assets provide social value through enhanced health-related quality of life (12). Celebration and meaning-making thus become integral to public health practice rather than peripheral concerns.

Collectively, these four orientations form the foundation of the Slow Public Health framework. This paradigm invites reconsideration of how health systems engage with aging populations affected by migration. Translating such a framework into practice requires institutional flexibility, revised evaluation criteria, and commitment to relational approaches. Slow Public Health's distinctive contribution lies not in its individual components, which are already evident in community-based participatory research and Slow Medicine, but in their integration at the population level. It also provides explicit guidance for calibrating between urgent and slow modes of response, rather than assuming deliberateness as a universal default. As a practical heuristic, urgent action should take precedence when there is immediate risk to safety or survival requiring time-sensitive intervention (e.g., acute illness, abuse, crisis, trauma). In contrast, the Slow register is more appropriate when outcomes depend on relational or process-based change that cannot be accelerated, such as building trust or repairing relationships with an isolated older adult. In practice, application typically entails dynamic calibration between these registers, rather than their treatment as mutually exclusive categories.

Table 1 summarizes the key distinctions between the conventional and Slow Public Health approaches across dimensions relevant to migration and aging contexts.

**Table 1:** Comparative overview of conventional public health and slow public health approaches in migration and aging contexts.

Dimension	Conventional public health	Slow public health
Temporal orientation	Project-cycle temporality; short funding cycles; rapid implementation and evaluation timelines	Life-course perspective; recognition of biographical, cohort, and community time; patience with long-term change
Community relationship	Communities as “intervention sites” to be targeted, measured, and evaluated	Communities as partners in health creation; longitudinal engagement built on trust
Engagement approach	Standardized programmes applied broadly; multiple interventions; efficiency prioritized	Deep listening before program design; one initiative at a time; quality of engagement over quantity
Outcome measures	Quantifiable metrics; disease reduction; behavior change rates; technology adoption	Quality of life; meaning-making; joy and contentment; strength of social connections
View of older adults	Service recipients; focus on deficits and needs; independence as ultimate goal	Contributors with assets and wisdom; focus on capacities; interdependence and reciprocity valued
Response to migration-induced care gaps	Replace absent caregivers with formal services; fill the “kinship vacuum” through programs	Understand meaning of loss first; mobilize existing community assets; build trust at sustainable pace
Underlying philosophy	More intervention yields better outcomes; speed and efficiency; standardization across contexts	Deliberate action over automatic response; presence over productivity; contextual adaptation
Practitioner Skills Emphasized	Program management; data collection; outcome measurement; scaling interventions	Deep listening; cultural humility; comfort with ambiguity; relationship building
Applicability in crises	Strong for urgent response and rapid risk reduction	Complementary; not a substitute for emergency response. Clarifies boundaries and avoids misuse of “slow” in emergencies.

Source: Own elaboration (2025).

The distinctions outlined in Table 1 are presented in idealized form for analytical clarity. Public health practice is rarely so neatly divided; in reality, elements of both approaches often coexist and may complement each other. The value of this comparison lies not in establishing rigid categories but in illuminating the different orientations and possibilities available in migration-related aging contexts. For example, in a community where grandparents care for grandchildren left behind by migrant parents, a conventional response might implement a scheduled visiting programme evaluated by the number of visits delivered. A Slow Public Health approach would first invest time in understanding existing support networks and then co-design services with the community. This example is illustrative rather than evaluative. Testing the framework across diverse settings remains an important priority for future research (see Section 6).

## 4 Asset-based approaches as methodological foundation

Asset-based approaches offer a methodological foundation for Slow Public Health that complements its philosophical orientation. Rather than asking, “what is wrong,” asset-based community development employs slow asset mapping that identifies what individuals and communities already possess (13); health assets, including personal, social, economic, and environmental factors, protect and promote health in older age (7).

Personal assets can be discovered through individual capacity inventories that identify skills accumulated over lifetimes. These include knowledge of local history and traditional practices, strengths such as resilience, wisdom, and caregiving capacity, and cultural and spiritual resources. The discovery process must be slow, guided by questions like: What brings you joy? What do you still want to contribute? (14). This example reflects a Singaporean urban setting. In other Global South settings, asset-mapping should be adapted to local languages, traditions, and social conditions rather than applied uniformly. For example, in Sub-Saharan Africa, older adults' assets are often rooted in indigenous knowledge and cultural heritage rather than in individual capacity inventories (15). In South Asian migrant-sending communities, unmet needs among older adults frequently relate to involve emotional and practical support, despite regular digital contact with migrant children (16). These differences highlight that both the questions asked and the assets identified should be locally grounded.

Social assets require slow relationship building that charts existing connections while identifying potential new ones. Crucially, this must include reciprocal relationships, examining not only who helps older adults, but who they help. Asset-based community development recognizes that without the contribution of older citizens, the work needed to activate health-promoting conditions will remain incomplete (17).

Economic assets, viewed through a Slow lens, extend beyond individual financial resources to include collective resources, structural support, and non-monetary exchange. Environmental assets include accessible public spaces designed with, rather than for, older adults and green spaces that support social connection (7, 14).

Asset-based approaches are not a substitute for structural support and formal service provision. Rather, they represent a complementary lens that helps identify what exists, what can be mobilized, and where formal systems must fill gaps. Without this clarity, asset-based language risks becoming a justification for withdrawing services in contexts where poverty, inequality, and systemic neglect create profound resource constraints. In this framework “slow” means intentional depth through sustained relational investment, not reduced, delayed or withheld care. Where “slower” approaches result in diminished access, quality, or responsiveness—particularly for already disadvantaged populations—they represent a misapplication of the framework rather than its intended use.

## 5 Slow public health in migration and aging contexts

For older adults affected by migration, whether left behind or having migrated themselves, Slow Public Health offers distinctive contributions. The disconnection created by migration disrupts traditional care networks and temporal rhythms, creating what might be termed a “kinship vacuum” that digital means can only partially address (18). Conventional public health responses often fill this vacuum through service provision; Slow Public Health suggests an alternative.

Rather than rushing to replace absent family caregivers with formal services, Slow Public Health would begin with deep listening to understand how affected older adults experience and make meaning of their situations (19). It would address the temporal dimensions of migration-induced changes, recognizing that adaptation may occur too slowly for standardized interventions. It would mobilize existing community assets while building new relational networks slowly enough for trust to develop (9).

The use of digital means for kinship and care provision highlights differences between conventional and Slow approaches. Conventional public health would measure success by technology adoption rates. In contrast, Slow Public Health would focus on the quality of connections maintained (18).

In the Global South context, Slow Public Health offers a framework that honors both the urgency of demographic transitions and the need for durable approaches. The concept of collective existence, central to many Southern cultures, aligns naturally with Slow Public Health's emphasis on interdependence and reciprocity rather than individual independence alone.

## 6 Discussion

The transition from conventional public health to a Slow Public Health approach represents not merely a methodological adjustment but a paradigm shift in how the relationship between time, health, and human flourishing is understood. This shift holds particular significance for the Global South, where the simultaneous challenges of youth migration and population aging demand sustainable, contextually appropriate responses.

Several implications emerge from this perspective. For policy, Slow Public Health suggests the need to reconsider funding cycles and evaluation timelines that fav or rapid, measurable outcomes over sustainable community capacity-building (9, 12). Longitudinal engagement requires commitments beyond typical project timelines. For practice, developing skills such as deep listening, cultural humility, and comfort with ambiguity, should be emphasized in conventional public health education (7, 11).

For research, Slow Public Health suggests methodological approaches that honor temporal depth, including longitudinal designs, narrative methods, and community-based participatory research that positions communities as genuine partners rather than research subjects (5, 10). The conceptual framework of settings, populations, and time proposed by Aagaard-Hansen and Bloch (6) offers a useful foundation for such research. Future work should test the Slow Public Health framework empirically across diverse Global South settings, using mixed methods and realist evaluation to clarify what works, for whom, and under what contextual conditions.

Slow Public Health also faces challenges. In contexts of acute need, calls for deliberation may seem inadequate. In genuine emergencies, rapid public health response must prevail. Resource-constrained health systems may struggle to justify approaches without rapid, measurable returns. The global health architecture, with its emphasis on metrics and accountability, may resist paradigms that prioritize process over outcomes (20). Yet these pressures, and demands for faster responses, greater efficiency, and measurable impacts, may contribute to the unsustainability of conventional approaches.

The path toward sustainability in addressing migration and aging may require a return to beginnings, a recognition that human health is fundamentally relational, temporal, and contextual. Slow Public Health offers not a rejection of progress but a reconsideration of what progress means: closer attention to communities' and individuals' histories and present realities.

In conclusion, Slow Public Health represents a viable and necessary paradigm for addressing the interconnected challenges of migration and aging in the Global South. By embracing life-course perspectives, longitudinal engagement, patience with outcomes, and attention to joy and meaning, this approach offers a road toward sustainability that conventional public health paradigms may struggle to provide. The challenge lies in transforming these principles into practice, a task that will itself require the deliberate, thoughtful, and system-oriented engagement that Slow Public Health advocates.

## Data Availability

The original contributions presented in the study are included in the article/supplementary material, further inquiries can be directed to the corresponding author.
